# A protocol for identifying the needs related to drug use, health and social (re)integration of people living in prison within five European countries

**DOI:** 10.1186/s13690-024-01405-2

**Published:** 2024-10-08

**Authors:** Els Plettinckx, Nadine Berndt, Rita Seixas, Stefaan De Smet, Jérôme Antoine, Helena Bruggeman, Nina Harth, Athanasia Papadopoulou, Ioulia Bafi, Anastasios Fotiou, Evelina Pridotkienė, Rima Kalinauskaitė, Brigita Rašimaitė, Myria Tsiakkirou, Margot Balcaen, Kim Fernandez, Lies Gremeaux, Nicky Dirkx, Karin De Ridder, Ioanna Yiasemi, Josefina Mavrou

**Affiliations:** 1 Sciensano, Rue Juliette Wytsmanstraat 14, Brussels, 1050 Belgium; 2 Direction de la santé - service d’épidémiologie et statistique, rue de Bitbourg 20, Luxembourg-Hamm, 1273 Luxembourg; 3 Research Centre SUPRB-Substance Use and Psychosocial Risk Behaviours, HOGENT University of Applied Sciences and Arts, Voskenslaan 364A, Gent, 9000 Belgium; 4 University of mental health, neurosciences and precision medicine research institute ‘Costats Stefanis’, Soranou tou Efesiou 2, 115 27, Athens, Greece; 5 Drug, Tobacco and Alcohol Control Department, Šv. Stepono str. 27A, Vilnius, 01312 Lithuania; 6 Prisoners rights protection, Larnaca, Cyprus; 7 Cyprus National Addictions Authority, 35 Iosif Hadjiosif and Andreas Avraamides, Strovolos, Nicosia, 2028 Cyprus; 8 Republican Centre for Addiction Disorders, Gerosios Vilties str. 3, Vilnius, 03147 Lithuania

**Keywords:** People living in prison, Drug use, Health, Social (re)integration needs, Mixed methods, Europe, Study protocol

## Abstract

**Background:**

Compared to the general population, people living in prison are at an increased risk to experience negative (mental) health outcomes. Moreover, delinquency and drug use have many risk factors in common. A need exists for increasing the knowledge about health needs, drug use patterns and the coverage of drug-related interventions in prison within Europe. The current protocol describes the design of a study about wellbeing, drug use and related care in prison.

**Methods/design:**

A multicentre mixed method design is implemented in five European countries (Belgium, Cyprus, Greece, Lithuania and Luxembourg). Qualitative and quantitative data collection tools are combined in order to generate complementary and comprehensive results. First, a cross-sectional survey among people living in prison is conducted. This survey is based on a model questionnaire, the European Questionnaire on Drug use among people living in Prison, developed by the European Monitoring Centre for Drug and Drugs Addiction. Second, people living in prison and people who have been recently released from prison are involved in qualitative semi-structured face-to-face interviews. The main topics of interest are the use of drugs and other health related topics such as loneliness, anxiety, depression, infectious diseases, suicide and treatment. Third, data regarding health and social reintegration measures in prison is collected through a quantitative survey addressed to the prison authorities.

**Discussion:**

This study protocol presentes a European study which aims to assess drug use among people living in prison and recently released people who use drugs as well as the existing care services in prisons. Hereby, factors related to the prison environment and their needs, both inside and outside prison are taken into account. Importantly, this study protocol describes a methodology which is developed to be executed in different prison settings within different countries simultaneously. Accordingly, for each country the protocol is adjustable to specific national legal requirements, regional differences and distinct local regulations of prison administrations. However, extensive modularity inevitably comes with significant limitations of comparability and generalizability of the results.

**Table:** 

**Text box 1. Contributions to the literature**
• This study protocol addresses the critical and often overlooked issue of health and drug use among people living in prison. Compared to the general population, people living in prison are at an increased risk to experience negative (mental) health outcomes, necessitating extensive research and targeted interventions.
• Given the overlapping risk factors for delinquency and drug use, this study protocol addresses a pressing public health need.
• This study protocol is designed to provide a robust framework that can be used by other researchers in order to conduct similar studies, offering flexibility while maintaining methodological consistency.

## Background

The estimated number of people living in prison (PLIP) in the European continent is 1,467,499 for 2019, meaning that 166.2 persons per 100,000 citizens are held in prison [1]. Overall, an increased risk exists to initiate drug use or the use of other substances once people are admitted to prison. The lifetime prevalence of illicit drug use of PLIP prior to prison is on average 61% in Europe [2]. Drug-related offences are the most common reason for which PLIP are sentenced to prison (excluding other unspecified offences) [3]. Worldwide, it is estimated that 23.8% (95% CI 21.0–26.7) of people meet diagnostic criteria for alcohol use disorder on arrival to prison, and 38.9% (31.5–46.2) for drug use disorder. Half of PLIP with major depression or psychotic illness also have a comorbid substance use disorder [4]. Cannabis is most frequently reported, followed by powder cocaine. Nevertheless, some studies report the highest prevalence of use for crack cocaine among newly admitted PLIP [2].

Compared to the general population, PLIP are at an increased risk to experience negative (mental) health outcomes such as cognitive impairments, (non-)communicable diseases and drug use [5, 6]. More PLIP have experienced injecting drug use compared to people without a prison history [5]. Moreover, delinquency and drug use have many risk factors in common, including social vulnerability, economic deprivation, school dropout, unemployment, child neglect and suicide [2, 7–13]. Consequently, these people have health care needs that require specific attention when entering prison, during detention and after release. Providing continuity of care as people move between prison and the community is key in achieving sustainable and effective treatment outcomes, and it is likely to have a significant impact on public health within the broader society [2, 4]. Accordingly, prisons and the criminal justice system are indispensable in the chain of drug-related interventions including prevention and treatment [5, 14].

Despite the abovementioned context, there remain significant differences in prevalence by country. Therefore, a need for additional research about health related needs and interventions among PLIP and recently released people who use drugs in European countries exists [2, 4]. The current study protocol is developed within 5 different countries, namely Belgium, Cyprus, Greece, Lithuania and Luxembourg. Among these countries, previous published research within prison is limited [2]. The current protocol describes a study to be conducted among these five countries with the primary objective to assess patterns of drug use and related problems in prison. In addition, the study protocol describes three secondary objectives. First, exploring drug use and related needs among PLIP. Second, studying drug use and related needs during detention among recently released PWUD. Third, describing drug-related interventions in selected prisons. This study enhances the monitoring and research in prisons and aims to generate knowledge about drug use and the accessibility of prevention, treatment and social reintegration interventions in prisons which is essential to design planning instruments to overcome drug use and reduce related morbidity or mortality. It also aims to generate knowledge on possibilities to harmonise the data collection among different prison settings. The study has started in 2021 and has a timespan of three years.

## Methods/design

### Study design

The current study consists of a multicentre mixed method design jointly conducted in Belgium, Cyprus, Greece, Lithuania and Luxembourg. Qualitative and quantitative data are collected in order to generate complementary and comprehensive results. The integration process of this design occurs during data collection, analysis and the presentation of results [15]. The study comprises three different components. One component consists of a cross-sectional survey among PLIP. A second component consists of qualitative semi-structured face-to-face interviews among PLIP and people who have been recently released from prison. A third component, which is the prison facility survey, gathers data about service provision through a quantitative survey addressed to the prison administration and specific prison departments.

### Setting and target population

PLIP have health care needs that require specific attention when entering prison, during detention and after release [2]. For this reason, both people in community and in prisons are targeted by the current study. Belgium and Greece are the countries with the largest general population involved in the study, followed by Lithuania, Cyprus and Luxembourg (Table 1). At the time of the data collection, the number of prisons ranges in the participating countries from 1 in Cyprus to 35 in Greece. The size of the prison population ranges from about 560 in Luxemburg to about 11,200 in Greece. The lowest prison rate per 10,000 population is observed in Cyprus [37] and the highest in Lithuania (186). The prison density, which is the ratio between the total number of PLIP and the total capacity of prisons in a country, is the lowest in Lithuania (72.6) and the highest in Greece (114.4) [16].

**Table 1:** Number of prisons and total population in the participating countries in 2021 [3]

	General population	*N* Prisons	Prison population	Prison population rate per 100,000 inhabitants	Prison density per 100 places
Belgium	11,522,440	34	10,374	90	108.4
Cyprus	888,005	1	571	37	110.5
Greece	10,718,565	35	11,266	106	114.4
Lithuania	2,794,090	8	5,188	186	72.6
Luxembourg	626,108	2	557	88	78.3

The mean age of the PLIP in the different countries varies between 37 and 39 years old and 95% of the prison population is male, which is in line with the average age and gender distribution in the European prisons [16]. As data is collected within prisons and in community in order to reach the research objectives, two target groups are envisaged, namely (a) PLIP and (b) people recently released from prison.

- PLIP are defined as every person aged 18 or older serving a sentence or being on remand in a closed prison in the participating countries during the period of the data collection. This definition excludes (i) juveniles, (ii) people who committed a crime and are held in open prisons, other institutions than penal institutions or those for whom an alternative to imprisonment is applied, (iii) PLIP with mental illness who have committed a crime but who are deemed to have not being responsible for their behaviour and (iv) PLIP in isolation for health reasons (e.g. due to Covid-19) or security issues. Consequently, juvenile detention centres and open prisons are excluded from this study. In addition, some participating countries also excluded (i) illiterate PLIP (in Luxembourg and Cyprus), (ii) people sentenced to life imprisonment (in Cyprus) and (iii) people sentenced because of sex offences (in Cyprus) due to their difficult accessibility and safety reasons. The corresponding sample size of PLIP is estimated within the framework of the quantitative data collection. It is determined for every participating country separately according to the prison population size of 31st of January of 2021 as described in Table 1. In addition to the prison population size, a margin of error of 5% and the confidence interval of 95% are taken into account [17]. This results in a sample size calculation of 228 respondents for Luxembourg, 230 respondents for Cyprus, 358 respondents for Lithuania, 372 respondents for Greece and 371 respondents for Belgium. In relation to the interviews, a convenience sample of 10 PLIP is defined.
- The inclusion criteria of people recently released from prison are (i) being ≥ 18 years of age, (ii) being released from prison within the past 12 months and (iii) having a history of drug use. Exclusion criteria are language limitations and mental or cognitive impairments that would impede participation in a semi-structured interview. A convenience sample of 10 participants per country is defined.

People recently released from prison are involved in the qualitative part of the research to better understand the impact of drug use, health and security related needs during detention and on reintegration into the open community.

### Stakeholders’ involvement

In each country, partnerships are established with both prisons and low-threshold community care initiatives in order to recruit respondents. Therefore, individual informative meetings between the researchers from each country on the one hand and the prisons and low-threshold community care initiatives on the other hand are organised to explain the aims of the study, its procedure, the data collection tools and methodologies (survey and interview guidelines). The detailed procedures to guarantee confidentiality and protection of the participants and their data are discussed as well. A safe context during data collection is the first priority in order to avoid both refusals and socially desirable answers due to fear of retaliation. Therefore, any kind of possible pressure and interference of third parties, e.g. prison staff, are prevented to the maximum extent possible. In this respect it is agreed that only aggregated data and final results are to be shared with the stakeholders. In Lithuania and Belgium, the data collection is fully performed by independent researchers. In Luxembourg, the survey in prison is conducted by staff from the somatic infirmary and the psychiatric infirmary. In Greece, a collaboration is established between independent researchers and social care services of the prisons. In Cyprus, the coordinator of the education department of the prison and custodial staff are involved in the data collection. These involved staff are bound by professional secrecy. Separate trainings concerning the procedure of data collection are specifically organised for the infirmary staff in Luxembourg. It involves (i) the procedure of informing participants about the study and its objectives, (ii) the procedure of conducting the survey and (iii) practical arrangements to conduct the interview, the provision of the written informed consent form, and the interview guide.

### Data collection

#### Quantitative cross-sectional survey

**Measures**

The data collection of the survey among PLIP is based on the European Model Questionnaire on Drug use in People living in prison (EQDP) developed by the European Monitoring Centre for Drugs and Drug Addiction (EMCDDA) [18]. The data collected by the EQDP is based on self-report. The questionnaire is fully anonymous and contains a set of variables that have to be mandatorily included in the survey to guarantee harmonization of the data collected among countries. This set of variables contains questions about the (i) socio-demographic characteristics, (ii) prison context, (iii) substance use (including alcohol, illicit drugs, prescription drugs that are not prescribed by a doctor, and injecting drug use) outside and inside prison, (iv) health outcomes that may be related to drug use such as mental health issues, overdoses, infectious diseases or suicide, (v) availability and their use of treatment interventions in prison and (iv) availability and their use of social reintegration interventions by PLIP (Table 2). A few additional topics are optional to integrate in the survey at national level.

**Table 2:** List of mandatory topics included in the survey among PLIP in all participating countries

Socio-demographic characteristics	Substance use outside and inside prison
Year of birth	Prevalence of substance use
Gender	Frequency of use
Housing	Injecting drug use
Household composition	Sharing (injection) equipment
Employment status	Onset of substance use during detention
Highest education level	Occurrence of overdose
**Prison context**	**Treatment in prison**
Prison history	Treatment visit
Duration of detention	Prescription of medication
Detention status	Drug related treatment
**Health**	Availability of interventions
Mental health	**Social reintegration**
Overdose	Housing
Infectious diseases	Housing conditions
Suicide	Employment

Within the survey, the current detention period is defined as a reference period. A list of 18 substances is covered within the survey including alcohol, cannabis, cocaine, crack, (meth)amphetamine, ecstasy, ketamine, hallucinogens, GHB/GBL, heroine, other opioids, sedatives, inhalants, new psychoactive substances, anabolic steroids and other substances. Individuals are defined as having used any drug when they respond positively to one of the selection questions concerning drug use before or during the current detention period. Accounting for differences in origin of PLIP both across and within countries, the survey is translated in 15 languages, including Lithuanian, Greek (including Greek Cypriot), English, French, German, Dutch, Portuguese, Turkish, Albanian, Russian, Arabic, Italian, Spanish, Polish and Romanian. The translations are conducted by professional translators and are doubled checked by native speakers afterwards. Every respondent can choose in which language they want to fill out the survey. An overview of the available languages in the specific countries is given in Table 3. The completion of the survey is estimated to take between 20 and 40 min.

**Table 3:** Availability of language choices of the survey among PLIP by participating country

	Participating countries				
	Belgium	Cyprus	Greece	Lithuania	Luxembourg
Lithuanian				x	
Greek		x	x		
English	x	x		x	x
French	x	x			x
German	x				x
Dutch	x				
Portuguese	x				x
Turkish	x				
Albanian	x		x		
Russian	x		x	x	
Arabic	x		x		
Italian	x				
Spanish	x				
Polish	x				
Romanian	x				

The last component of the study is the prison facility survey which is based on the European Facility Survey Questionnaire on Prisons (EFSQP) developed by EMCDDA. This survey among the prison administrations enables to draw a baseline to indicate what interventions exist in the participating prisons over a period of one year. The questionnaire contains questions about the prison characteristics, yearly statistics of the prison population, availability of drug-related interventions or treatment, drug testing, involvement of prison staff within these interventions and quality assurance measures (Table 4). This facility survey is made available in the official languages of the countries concerned.

**Table 4:** List of topics included in the facility survey in the participating countries

**Prison characteristics**	**Drug tests**
Name of the prison	Performance of drug tests
Type of the prison	Type of drug tests performed
Available sections in the prison	Number of people tested
**Prison population**	Total number of drug tests
Capacity of the prison	Number of positive tests by substance
Average daily number of people in prison (by age, sex and status)	Consequences of a positive drug test
**Drug-related interventions**	**Prison staff in drug-related interventions**
Availability of the interventions	Type of prison staff involved
Capacity of the interventions	Number of prison staff involved
Number of people accessing the interventions	**Quality assurance**
Screening of health-related aspects	Mechanisms for quality control
**Drug treatment**	Trainings about drug-related interventions
Total number of people in treatment	**Contact details**
Number of people in treatment by substance	e-mail address responding prison service

**Recruitment procedures**

In Luxembourg, Lithuania, Greece and Cyprus, a non-probability, purposive sampling approach (convenience sample based on self-nomination) is used to recruit PLIP (Table 5). A random sampling based on the list of PLIP is executed in Belgium. Because of the specific Belgian situation, prisons in each of the three regions of the country are represented: (i) Flanders (Dutch-speaking), (iii) Brussels (Dutch-French speaking) and (iii) Wallonia (French speaking). In line with the approval of the prison administration, a brief introduction about the research project is provided orally to the eligible PLIP by the researchers or the prison staff in Belgium, Cyprus, Greece and Lithuania. In Luxembourg, PLIP are informed by flyers and posters distributed to all PLIP a few days before the data collection took place. Both approaches allow eligible PLIP to ask questions before their actual decision to participate in the study. Participants have to agree with an informed consent before starting the survey. In Cyprus, Greece, Luxembourg and Lithuania, the self-administered questionnaire is filled out in a pencil-paper format. In Belgium, the survey is administrated digitally on tablets [19]. The latter increases the confidentiality and autonomy because questions are presented gradually on the screen and automatic consistency checks are executed whereby inconsistencies could be corrected immediately. The questionnaire is made available either individually in each cell (in Luxembourg), in a separate private room for sequential individual completion (in Greece) or in groups of 5 to 15 people in a separate room for individual completion (in Belgium, Cyprus and Lithuania). The EU General Data Protection Regulation is addressed in order to guarantee confidential and voluntary participation. Participants of the pencil-paper questionnaires are asked to hand the completed questionnaire in one envelope, to close the envelope and to deliver it in one box that do not allow seeing or taking the envelopes without having to destroy them. In addition, they have to hand the informed consent form in another envelope, to close this envelope as well and to deliver it separately in a different closed box. PLIP can keep the study information sheet. In Belgium and Greece, structured face-to-face interviews are conducted in case the PLIP have issues with reading or in case difficulties to fill out the questionnaire are observed. In Greece, mediators translate the questions orally in case PLIP are not familiar with the Greek language. In Cyprus, Belgium, Greece and Lithuania, all respondents who complete the survey get a small incentive (e.g. towel). In Luxembourg, the permission to give an incentive is not obtained.

**Table 5:** Recruitment procedure of the survey among PLIP by participating country

	Participating countries				
	Belgium	Cyprus	Greece	Lithuania	Luxembourg
**Information about the survey**					
In cell by researcher or prison staff	x	x	x	x	
Flyers					x
**Sampling**					
Non-probability sampling		x	x	x	x
Random sampling	x				
**Presence of researchers**					
Availability of researchers for further questions	x	x	x	x	x
**Administration of the survey**					
Pencil-paper survey		x	x	x	x
Digital survey	x				
**Place of completion of the survey**					
Survey completion in cell					x
Survey completion in separate room	x	x	x	x	
**Participation**					
Guarantee of confidential and voluntary participation	x	x	x	x	x
Incentive	x	x	x	x	

The prison facility survey developed by EMCDDA is made available for each prison in which also PLIP are involved in the study. This facility survey is made available either digitally or on paper format. Only one survey per prison has to be filled out. The survey can be completed by several staff members accordingly to their expertise. The questions in both quantitative surveys are not mandatory which allow the respondents to skip one or more questions.

#### Qualitative semi-structured interviews

**Measures**

The qualitative interviews in the participating countries are conducted by a semi-structured interview guide. Topics are designed to explore the individual perspectives and needs of the participants, including health, psycho-social wellbeing, substance use before imprisonment, substance use during imprisonment, motives of use and the context of substance use, drug-related needs and the possibility of getting support or treatment. The interviews are planned to last about one hour. The interviews can be conducted in a (limited) number of languages, according to the interviewers’ linguistic competences (Dutch, English, French, German, Greek and Lithuanian).

**Recruitment procedures**

Only PLIP who complete the survey and who reports drug use during imprisonment are eligible to participate to the qualitative semi-structured interview. PLIP can indicate to be willing to participate in the interview. Those interested are contacted shortly afterwards to arrange the day and time of the interview. The recruitment of recently released people who used drugs (PWUD) (< one year) occurs in Belgium and in Luxembourg by flyers and posters in low-threshold community care initiatives. In Lithuania, Greece and Cyprus, NGO’s and probation workers are involved as mediators to bring PWUD in contact with the research teams. Both PLIP and PWUD have the opportunity to ask further questions before they engage in the interview. The respondents are asked to agree with an informed consent before starting the interview. In Belgium, the interviews are recorded, transcribed and analysed. In the other countries voice recordings are not allowed and the researchers take notes while guarantying cofidentiality and anonymity of the provided answers. In accordance with the survey, all respondents, except in Luxembourg, receive a small incentive as well.

### Analytic plan

#### Quantitative data analysis

Per country, the generated data is entered in a national database. Concerning the pencil-paper questionnaires, the responses are first compiled by scanning the survey forms or by manually entering the responses into a national database [20]. In the database, each record corresponds to an individual respondent. Quality checks to inspect for multiple and partial responses are performed, yielding higher quality data [18]. Based on primary outcome measures, the questions about ever drug use outside and inside prison are defined as key questions. In case respondents do not answer these key questions, these respondents are not taken into account for further data analyses. Consistency checks are conducted. For example, when some of the answers for the use of specific drugs are missing, but at least an answer is given for one of these drugs, it is assumed that the missing value is ‘never used’. When the prevalence of all these drugs is missing, these answers remain missing. Once the data are cleaned, new variables are created. The data is analysed for the different countries separately. In order to analyse the quantitative data coherently among the different countries, a common codebook is developed and used. No individual respondent can be identified based on the analyses. The location of the prison is not taken into account within the data-analyses. Variables such as age and duration of the detention are aggregated in broader categories.

#### Analyses of the semi-structured interviews

When semi-structured interviews are audio-recorded (Belgium) than the content is transcribed verbatim first. In case no voice recordings (other countries) are available, the notes taken during the in-depth interviews are completed immediately after each interview. This is done preferably on the same day in order to minimise recall bias [21]. Based on these transcriptions and notes, the research teams of the different countries use iterative and simultaneous content analysis to identify themes and key codes within the data. Comparative analysis and multiple coding are conducted. Text fragments from several interviews are chosen and coded according to an initial code structure. It should be noted that a first version of the coding structure is based on the Belgian data since these had been recorded and transcribed verbatim. The meaning and content of codes are discussed thoroughly. To ensure a high level of inter-coder reliability, a consensus between coders is sought on an ongoing basis. The coding structure is designed to be simple and straightforward [22]. These qualitative results are used to interpret and refine the survey findings within the context of lived experiences of participants. This approach is proven effective in generating an understanding of the social dimensions underpinning quantitative outcomes [23, 24].

## Discussion

This protocol and rationale of the current study are an important contribution to the literature because they provide full study transparency and useful information for researchers who plan to conduct a similar study in the future. To the best of our knowledge, this research is the first to study commonly drug use and related topics among PLIP in five different countries within a single methodological framework and time period. Previous published research within prison among these countries is limited. The triangulation of quantitative and qualitative data increases available evidence on (i) the prevalence of drug use before and during detention, (ii) the harms and needs of PLIP and PWUD regarding health and drug-related problems, and (iii) the interventions targeting them. Consequently, the obtained results can eliminate knowledge gaps and raise awareness on the importance of continued and harmonized monitoring of drug use and health aspects in prison settings in relation to treatment and social reintegration needs [25–27]. Appropriate measures should be taken within the prison environment to specifically assure the health and well-being, as well as the social (re)integration and security of PLIP who use drugs. Although the mean prison density per 100 places is decreasing from 99.1 in 2011 to 84.8 in 2022 among the Council of Europe member states, overcrowding is still a reality in several countries which makes the implementation of social and health related interventions even more difficult in the prison setting [3, 28–35]. This study protocol provides a description about the implementation of drug research in prison that can be implemented among other national communities and countries. Consequently, the results generated by the current study contribute to the improvement of existing prison healthcare programs by triangulating data from the period before, during and after detention. This protocol enables a more effective, innovative and agile approach to the growing complexity of health and drug use among PLIP. Moreover, its expected results aim to increase the preparedness to respond to future challenges and crises in the EU member states [36]. These practices can be a starting point for developing innovative actions within prisons and improve the health situation among countries. This information is socially significant because prisons present an opportunity to treat drug use and related health needs [13, 28]. The provision of treatment is associated with a range of positive outcomes which improve both the health who experience detention and the health of the community in which to return. This, in turn, contributes to the equivalence of care which is one of the United Nations’ Standard Minimum Rules for the Treatment of Prisoners, the Nelson Mandela Rules [37]. It is essential to reduce health inequalities within prison and in community.

The described study protocol targets a maximum harmonisation. Nevertheless, the specific conditions applied in the countries make comparison difficult. It is well-known that the conditions of data collection in prisons require more adaptability of researchers and prison staff, than equivalent research activities in non-prison environments [2, 38]. In this European project, the heterogeneity of conditions between the participating countries is caused by specific legal and practical circumstances. Limitations concerning the implementation of the study and the data collection are inevitable. First of all, the unique setting of a prison affects the course of the data collection. Researchers are dependent from the participation of prisons. They have to negotiate the way in which the initial introduction and communication about the research to the respondents occurs. Also, little flexibility exists in the timing and setting in which the research takes place (e.g. available rooms, background noise, security checks, respondent availability, etc.). Secondly, the results are hardly generalisable due to the convenience sample that is mostly applied due to the specific context of the prison setting and recruitment of PLIP in research studies. A limitation of convenience sampling is that only participants with certain characteristics present themselves to participate (e.g. a more stable psychological situation, a trustful relationship with the health team, a particular interest or sensitivity to the topic). This might result in a sample which do not represent the defined target population. In addition, we can also expect that not all eligible PLIP are reached due to work schedules or when PLIP are still sleeping in their cell when approached by the researchers. Moreover, the restrictions of the coronavirus pandemic are likely to influence the composition of the prison population because people with short-term prison sentences are released from prison during this period. Consequently, the proportion of long-term PLIP increases in the daily prison population [39]. Thirdly, even though the survey is translated in several languages and assistance for the respondents who have difficulties to read or write is available in different countries, it cannot be avoided that certain eligible people are not able to participate. Specifically, in relation to the interviews it is possible that not all of the participants who agree to be interviewed are interviewed. The knowledge of certain languages, the level of literacy or perhaps cognitive capacities of the respondents might have an impact on the final results. As a result, the risk of selection bias should be taken into account. Fourthly, the study is prone to response bias because of the self-reported nature of the collected data. This can be particularly due to social desirability as the restricted nature of the prison setting is challenging to protect the privacy and confidentiality of participants. Hence, a risk of over- and underestimation of certain outcomes exist [26, 27]. In some countries, this risk might be even bigger due to the presence of prison staff during the data collection [2]. Fifthly, as the majority of the questionnaires are filled out in paper-pencil format and because of the prohibition in most countries to you use voice recording within the prisons, this might have an impact on the data quality and efficiency. It is known that data collection through an electronic devise is more easy to handle which results in fewer errors [40]. Sixthly, recall bias or misinterpretation of the questions might have an impact on the results of the current study. In this respect comparability and generalizability of the results are restricted.

## Data Availability

Further documentation and information that is used or analysed during the current study (such as the questionnaire, the interview guideline, codebook of the database and decision tree) are available from the corresponding author on reasonable request.

## Abbreviations

PLIP: People Living in Prison
PWUD: People Who Use Drugs
EMCDDA: European Monitoring Centre for Drugs and Drug Addiction
EQDP: European Model Questionnaire on Drug use in Prisons
EFSQP: European Facility Survey Questionnaire on Prisons
NGO: Non-governmental organisation
EU: European Union
